# Supplementary material for: TMUB1 expression is associated with the prognosis of colon cancer and immune cell infiltration
Source: PeerJ. 2023 Nov 17;11:e16334. doi: 10.7717/peerj.16334 (PMC10658890; doi:10.7717/peerj.16334)
Supplement: Supplemental Information 1 — (A) Prognostic nomogram of TMUB1 based on overall survival. (B–D) Calibration curves of overall survival at year 1, year 3, and year 5. (E) Prognostic nomogram of TMUB1 based on OS. (F, G, H) Calibration curves of disease-specific survival at year 1, 3, and 5. [file peerj-11-16334-s001.pdf]

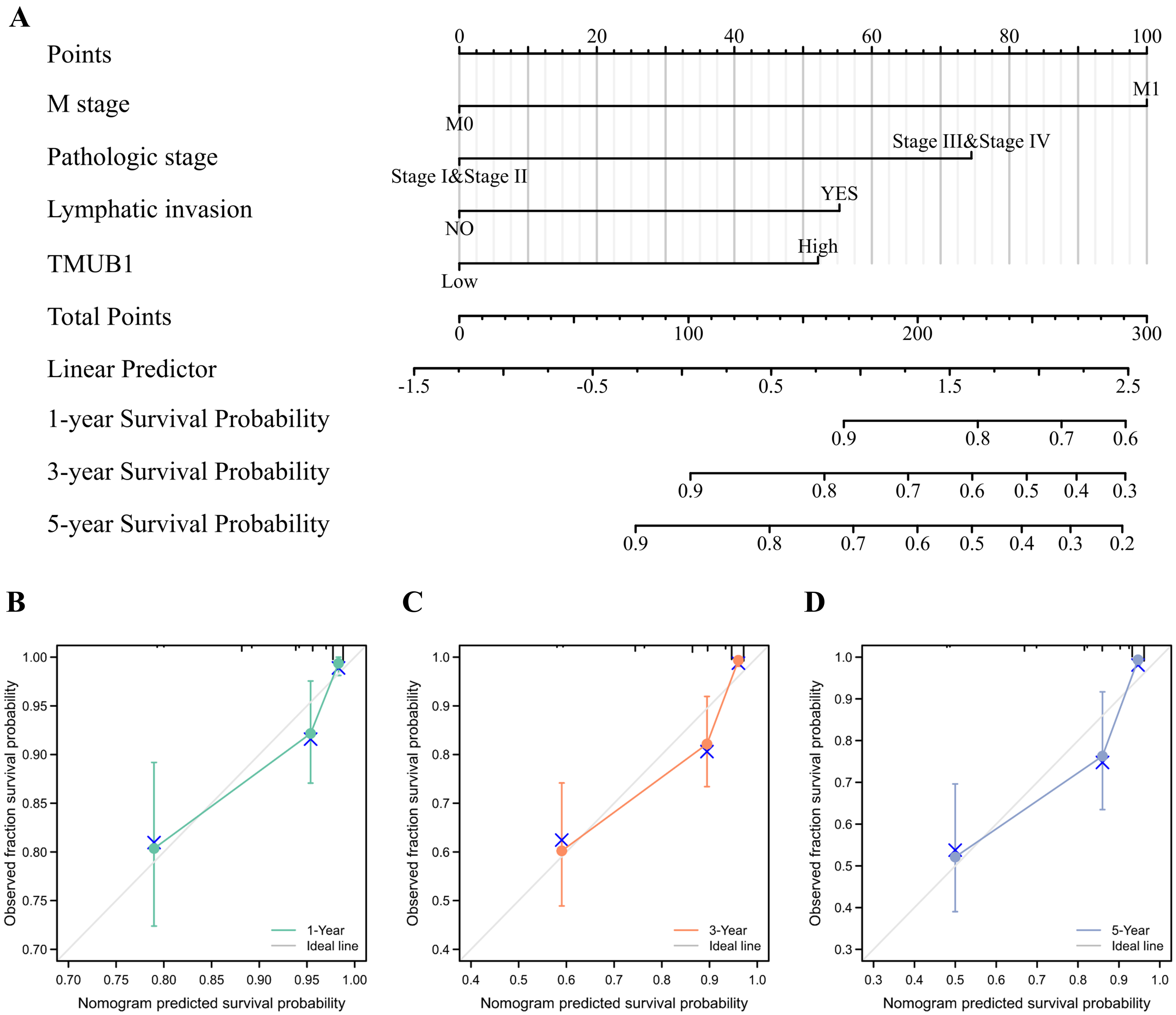
A
Points
M stage
Pathologic stage
Lymphatic invasion
TMUB1
Total Points
Linear Predictor
1-year Survival Probability
3-year Survival Probability
5-year Survival Probability
M0
M1
Stage I&Stage II
Stage III&Stage IV
NO
YES
Low
High
B
C
D
Observed fraction survival probability
Nomogram predicted survival probability
1-Year
3-Year
5-Year
Ideal line

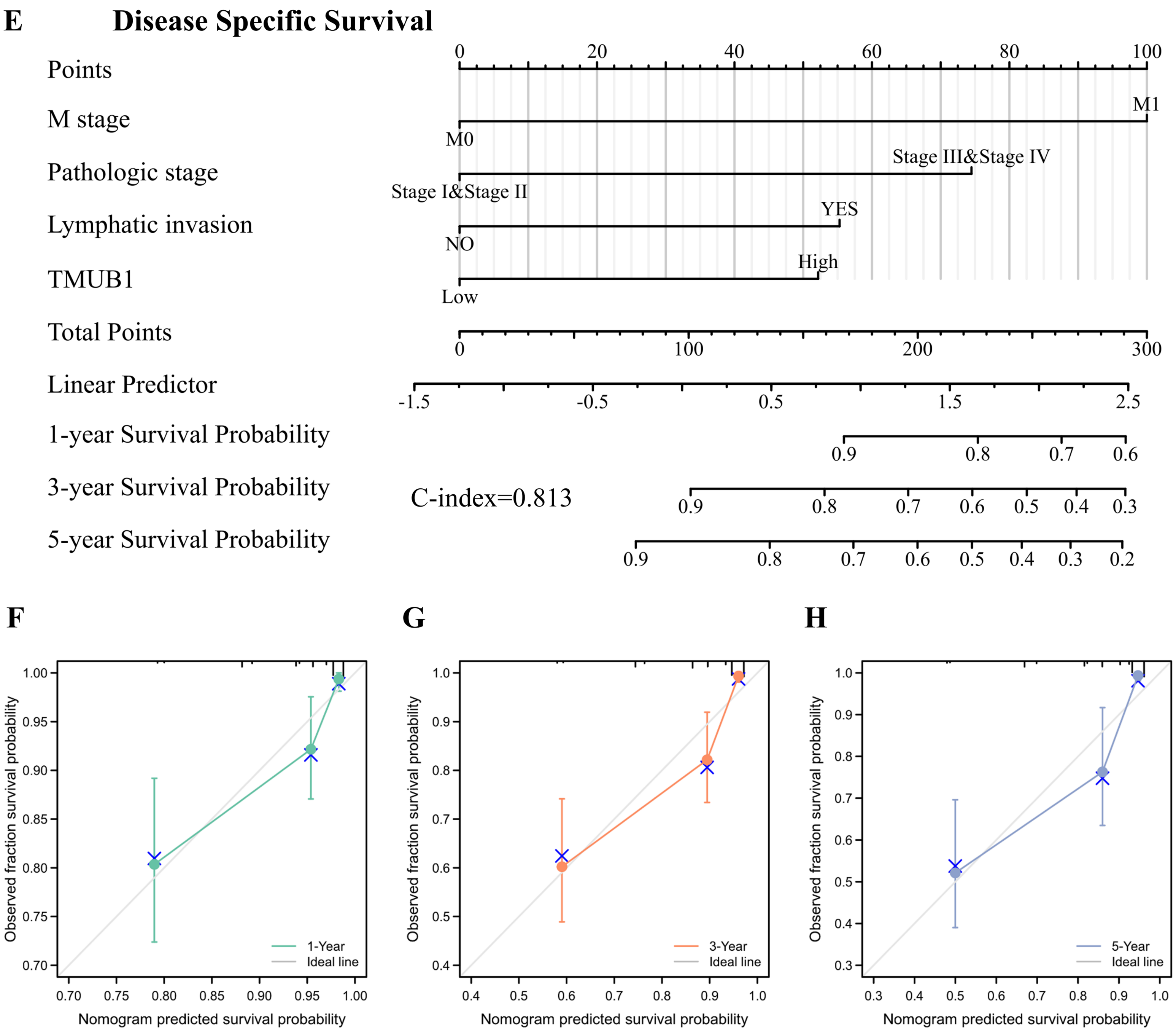
E
Disease Specific Survival
Points
M stage
Pathologic stage
Lymphatic invasion
TMUB1
Total Points
Linear Predictor
1-year Survival Probability
3-year Survival Probability
5-year Survival Probability
0 20 40 60 80 100
M1
M0
Stage III&Stage IV
Stage I&Stage II
YES
NO
High
Low
0 100 200 300
-1.5 -0.5 0.5 1.5 2.5
0.9 0.8 0.7 0.6
C-index=0.813
0.9 0.8 0.7 0.6 0.5 0.4 0.3
0.9 0.8 0.7 0.6 0.5 0.4 0.3 0.2
F
G
H
Observed fraction survival probability
Nomogram predicted survival probability
1-Year
3-Year
5-Year
Ideal line
