# Supplementary material for: TMUB1 expression is associated with the prognosis of colon cancer and immune cell infiltration
Source: PeerJ. 2023 Nov 17;11:e16334. doi: 10.7717/peerj.16334 (PMC10658890; doi:10.7717/peerj.16334)
Supplement: Supplemental Information 2 [file peerj-11-16334-s002.docx]

| Characteristics | Total(N) | Univariate analysis | |  | Multivariate analysis | |
| --- | --- | --- | --- | --- | --- | --- |
|  |  | Hazard ratio (95% CI) | P value |  | Hazard ratio (95% CI) | P value |
| T stage | 476 |  |  |  |  |  |
| T1&T2 | 94 | Reference |  |  |  |  |
| T3&T4 | 382 | 3.072 (1.423-6.631) | **0.004** |  | 3.332 (1.018-10.910) | **0.047** |
| N stage | 477 |  |  |  |  |  |
| N0 | 283 | Reference |  |  |  |  |
| N1&N2 | 194 | 2.592 (1.743-3.855) | **<0.001** |  | 0.343 (0.110-1.068) | 0.065 |
| M stage | 414 |  |  |  |  |  |
| M0 | 348 | Reference |  |  |  |  |
| M1 | 66 | 4.193 (2.683-6.554) | **<0.001** |  | 1.906 (1.064-3.414) | **0.030** |
| Pathologic stage | 466 |  |  |  |  |  |
| Stage I&Stage II | 267 | Reference |  |  |  |  |
| Stage III&Stage IV | 199 | 2.947 (1.942-4.471) | **<0.001** |  | 4.232 (1.239-14.458) | **0.021** |
| Lymphatic invasion | 433 |  |  |  |  |  |
| NO | 265 | Reference |  |  |  |  |
| YES | 168 | 2.450 (1.614-3.720) | **<0.001** |  | 1.771 (1.045-3.002) | **0.034** |
| *TMUB1* | 477 |  |  |  |  |  |
| Low | 239 | Reference |  |  |  |  |
| High | 238 | 1.778 (1.195-2.644) | **0.005** |  | 1.917 (1.176-3.124) | **0.009** |
