# Supplementary material for: TMUB1 expression is associated with the prognosis of colon cancer and immune cell infiltration
Source: PeerJ. 2023 Nov 17;11:e16334. doi: 10.7717/peerj.16334 (PMC10658890; doi:10.7717/peerj.16334)
Supplement: Supplemental Information 3 [file peerj-11-16334-s003.docx]

| Characteristics | Total(N) | Univariate analysis | |  | Multivariate analysis | |
| --- | --- | --- | --- | --- | --- | --- |
|  |  | Hazard ratio (95% CI) | P value |  | Hazard ratio (95% CI) | P value |
| T stage | 460 |  |  |  |  |  |
| T1&T2 | 93 | Reference |  |  |  |  |
| T3&T4 | 367 | 7.758 (1.896-31.745) | **0.004** |  | 2.173 (0.495-9.545) | 0.304 |
| N stage | 461 |  |  |  |  |  |
| N0 | 275 | Reference |  |  |  |  |
| N1&N2 | 186 | 4.059 (2.353-7.003) | **<0.001** |  | 0.299 (0.091-0.986) | 0.057 |
| M stage | 399 |  |  |  |  |  |
| M0 | 334 | Reference |  |  |  |  |
| M1 | 65 | 7.833 (4.597-13.346) | **<0.001** |  | 3.080 (1.562-6.073) | **0.001** |
| Pathologic stage | 451 |  |  |  |  |  |
| Stage I&Stage II | 259 | Reference |  |  |  |  |
| Stage III&Stage IV | 192 | 6.085 (3.235-11.447) | **<0.001** |  | 7.091 (1.758-28.606) | **0.006** |
| Lymphatic invasion | 422 |  |  |  |  |  |
| NO | 255 | Reference |  |  |  |  |
| YES | 167 | 4.133 (2.361-7.235) | **<0.001** |  | 2.579 (1.240-5.361) | **0.011** |
| *TMUB1* | 461 |  |  |  |  |  |
| Low | 233 | Reference |  |  |  |  |
| High | 228 | 2.231 (1.323-3.761) | **0.003** |  | 1.897 (1.011-3.560) | **0.046** |
