# Supplementary material for: TMUB1 expression is associated with the prognosis of colon cancer and immune cell infiltration
Source: PeerJ. 2023 Nov 17;11:e16334. doi: 10.7717/peerj.16334 (PMC10658890; doi:10.7717/peerj.16334)
Supplement: Supplemental Information 4 [file peerj-11-16334-s004.docx]

| TUMB1 | Transmembrane and ubiquitin-liked omaincontaining 1 protein |
| --- | --- |
| TCGA | The Cancer Genome Atlas |
| GEO | Gene Expression Omnibus |
| HPA | Human Protein Atlas |
| GO | Gene Ontology |
| KEGG | Kyoto Encyclopedia of Genes and Genomes |
| GSEA | Gene Set Enrichment Analysis |
| TPM | Transcripts Per Kilobase of exon model per Million mapped reads |
| FPKM | Fragments Per Kilobase Million |
| K-M | Kaplan-Meier |
| ROC | Receiver Operating characteristic Curve |
| DEGs | [Differentially Expressed Genes](http://www.youdao.com/w/differentially expressed genes%EF%BC%88DEGs%EF%BC%89/" \l "keyfrom=E2Ctranslation) |
| CEA | Carcinoembryonic antigen |
| OS | Overall Survival |
| DSS | Disease Free Survival |
| PFI | Progression- free interval |
| BP | Biological Process |
| CC | Cellular [Component](https://so.csdn.net/so/search?q=Component&spm=1001.2101.3001.7020) |
| MF | Molecular Function |
| COAD | Colon Adenocarcinoma |
| NATs | Normal Tissue Around cancer tissues |
| TPR | True Positive Rate |
| FPR | False Positive Rate |
