# Supplementary material for: TMUB1 expression is associated with the prognosis of colon cancer and immune cell infiltration
Source: PeerJ. 2023 Nov 17;11:e16334. doi: 10.7717/peerj.16334 (PMC10658890; doi:10.7717/peerj.16334)
Supplement: Supplemental Information 5 [file peerj-11-16334-s005.docx]

| Survival analysis | Kaplan-Meier analysis |
| --- | --- |
| Diagnostic analysis | Receiver Operating Characteristic (ROC) |
| Analysis of clinical prognostic significance | Single-multifactor Cox regression analysis |
| Prognostic prediction | Multifactor Cox regression analysis |
| Integrated differentially expressed Genes (DEGs) | R package: DESeq2 |
| Protein interaction Network (PPI) | R package: igraph |
|  |  |
